# RNA-Free and Ribonucleoprotein-Associated Influenza Virus Polymerases Directly Bind the Serine-5-Phosphorylated Carboxyl-Terminal Domain of Host RNA Polymerase II

**DOI:** 10.1128/JVI.00494-16

**Published:** 2016-06-10

**Authors:** Mónica Martínez-Alonso, Narin Hengrung, Ervin Fodor

**Affiliations:** a Sir William Dunn School of Pathology, University of Oxford, Oxford, United Kingdom; b Division of Structural Biology, University of Oxford, Oxford, United Kingdom; St. Jude Children's Research Hospital

## Abstract

Influenza viruses subvert the transcriptional machinery of their hosts to synthesize their own viral mRNA. Ongoing transcription by cellular RNA polymerase II (Pol II) is required for viral mRNA synthesis. By a process known as cap snatching, the virus steals short 5′ capped RNA fragments from host capped RNAs and uses them to prime viral transcription. An interaction between the influenza A virus RNA polymerase and the C-terminal domain (CTD) of the large subunit of Pol II has been established, but the molecular details of this interaction remain unknown. We show here that the influenza virus ribonucleoprotein (vRNP) complex binds to the CTD of transcriptionally engaged Pol II. Furthermore, we provide evidence that the viral polymerase binds directly to the serine-5-phosphorylated form of the Pol II CTD, both in the presence and in the absence of viral RNA, and show that this interaction is conserved in evolutionarily distant influenza viruses. We propose a model in which direct binding of the viral RNA polymerase in the context of vRNPs to Pol II early in infection facilitates cap snatching, while we suggest that binding of free viral polymerase to Pol II late in infection may trigger Pol II degradation.

**IMPORTANCE** Influenza viruses cause yearly epidemics and occasional pandemics that pose a threat to human health, as well as represent a large economic burden to health care systems globally. Existing vaccines are not always effective, as they may not exactly match the circulating viruses. Furthermore, there are a limited number of antivirals available, and development of resistance to these is a concern. New measures to combat influenza are needed, but before they can be developed, it is necessary to better understand the molecular interactions between influenza viruses and their host cells. By providing further insights into the molecular details of how influenza viruses hijack the host transcriptional machinery, we aim to uncover novel targets for the development of antivirals.

## INTRODUCTION

The segmented negative-sense RNA genome of influenza A virus is transcribed and replicated by the viral RNA-dependent RNA polymerase, which consists of three subunits, the polymerase basic 1 (PB1), PB2, and polymerase acidic (PA) proteins (1–3). Transcription and replication of the viral RNA genome are carried out in the context of viral ribonucleoprotein (vRNP) complexes in which the 5′ and 3′ termini of viral RNA (vRNA) interact with the viral polymerase, while the rest of the RNA is coated by nucleoprotein (NP) (4, 5). Influenza A virus is dependent on the host RNA polymerase II (Pol II) transcriptional machinery. Viral transcription requires 5′ capped primers, which are derived from host capped RNAs (6–9). Furthermore, active Pol II transcription is required for nuclear export of viral mRNAs (10). Previous studies by our group showed that Pol II coimmunoprecipitates with influenza A virus polymerase from infected cell lysates, and trimeric recombinant viral polymerase interacts with the serine-5-phosphorylated form of the C-terminal domain (CTD) of Pol II that is characteristic of initiating Pol II (11). Interaction between the viral polymerase and Pol II was confirmed by further studies (12–15). In addition, influenza virus polymerase was also shown to associate with Pol II promoter DNA (16).

Despite the clear functional and physical links between the viral and host transcriptional machineries, the details of this interaction remain poorly understood. In particular, it is not clear whether only free polymerase interacts with the CTD of Pol II or whether viral polymerase in the context of vRNPs can also interact. Although the influenza virus polymerase requires active Pol II to provide it with a source of capped RNA primers, the viral polymerase has also been linked to Pol II degradation. This occurs at late times during infection (17, 18), when free polymerase is present, and coincides with the shutdown of viral mRNA synthesis (18). Therefore, association of a free heterotrimeric polymerase with the CTD of Pol II might promote Pol II degradation, while binding of a fully assembled vRNP would more likely facilitate cap snatching by positioning the viral polymerase next to a supply of nascent, host capped RNAs. Additionally, it is also unknown whether the interaction between the viral polymerase and the Pol II CTD is direct or mediated by cellular factors. In fact, this issue remains controversial. While some reports point at cellular factors such as hCLE (19, 20) and cyclin T1/CDK9 (21) as mediators of the interaction between the viral polymerase and Pol II, other reports suggest that this interaction is direct (14). This study was designed to address these questions. Our results indicate that the viral polymerase can interact with the CTD of Pol II that is engaged in active transcription in RNA-free form, as well as in the context of vRNPs, raising the possibility that the interaction of the viral polymerase with Pol II could fulfill multiple functions.

## MATERIALS AND METHODS

### RIP.

RNA immunoprecipitation (RIP) was performed as previously described (16, 18, 22), with some modifications. Briefly, HEK 293T cells about 50% confluent were mock infected or infected with influenza A/WSN/33 virus at a multiplicity of infection (MOI) of 5. Cells were harvested at 4.5 h postinfection (hpi) and cross-linked with 1% formaldehyde for 10 min at room temperature, and the reaction was quenched by adding glycine to a final concentration of 125 mM. Cells were washed twice with cold phosphate-buffered saline (PBS) and lysed in buffer A (50 mM Tris-HCl [pH 8.0], 0.5% Igepal, 100 mM NaCl, 1 mM dithiothreitol [DTT], 1 complete mini EDTA-free protease inhibitor cocktail tablet [Roche]/10 ml of buffer). Cells were sonicated for 12.5 min with a Bioruptor (Diagenode), and cell lysates were clarified by centrifugation at 16,200 × *g* for 5 min. Cell lysates were supplemented with MgSO_4_ and CaCl_2_ to final concentrations of 10 and 1 mM, respectively, and treated with RNase-free DNase (Promega catalog no. M610A) for 30 min at 37°C. The reaction was stopped by adding EDTA to a final concentration of 20 mM. These samples were immunoprecipitated overnight at 4°C with antibodies specific for PA (kind gift from T. Toyoda) and Pol II (RNA Pol II, clone CTD4H8; Millipore catalog no. 05-623) and protein G-Sepharose (Sigma). Immunocomplexes were washed with 10 mM Tris-HCl (pH 8.0)–0.1% Igepal–1 mM phenylmethylsulfonyl fluoride (PMSF)–1 mM EDTA containing 150 mM NaCl (once), 1 M NaCl (three times), and 0.5 M LiCl (three times). Cross-links were reversed in both immunocomplexes and input samples by the addition of elution buffer (1% SDS, 50 mM Tris-HCl [pH 6.8], 200 mM NaCl, 1 mM EDTA) and heating at 65°C overnight. Protein G-Sepharose was removed by centrifugation, and samples were treated with proteinase K for 30 min at 45°C. RNA was extracted with phenol-chloroform and precipitated with ethanol in the presence of tRNA carrier. RNA samples were subjected to primer extension analysis of viral neuraminidase (NA) and NP segment-specific RNAs as previously described (23), except that products were analyzed on 6% polyacrylamide gels containing 7 M urea. The primers used were 5′-TCCAGTATGGTTTTGATTTCCG-3′ (for NA mRNA and cRNA), 5′-TGGACTAGTGGGAGCATCAT-3′ (for NA vRNA), 5′-ATCGTCCAATTCCACCAATC-3′ (for NP mRNA and cRNA), 5′-GAGCTCTCGGACGAAAAGG-3′ (for NP vRNA), and 5′-TCCCAGGCGGTCTCCCATCC-3′ (for 5S rRNA).

### Design and synthesis of Pol II CTD mimic peptides.

Peptides were chemically synthesized by Cambridge Peptides Ltd. by solid-phase peptide synthesis. Peptides were designed to contain four repeats of the heptapeptide consensus sequence of the Pol II CTD (YSPTSPS) with modifications representing different phosphorylation states of the CTD. Full amino acid sequences are shown in Table 1. All peptides were synthesized with C-terminal amidation and N-terminal biotinylation and contained a nine-atom polyethylene glycol spacer between the biotin moiety and the first amino acid. Peptides were purified by high-performance liquid chromatography to at least 90% purity. Peptide quality control was performed by mass spectrometry.

**TABLE 1:** Design of Pol II CTD mimic peptides with different phosphorylation states

Peptide	Sequence^*a*^
Ser2P	Y(pS)PTSPSY(pS)PTSPSY(pS)PTSPSY(pS)PTSPS
Ser5P	YSPT(pS)PSYSPT(pS)PSYSPT(pS)PSYSPT(pS)PS
Unphosphorylated	YSPTSPSYSPTSPSYSPTSPSYSPTSPS
Scrambled	PSSSTPSSYTPSPSSSPTSYSPYYTSPP

a (pS) represents phosphoserine.

### Pulldown assays with CTD mimic peptides.

HEK 293T cells were mock infected or infected with influenza A/WSN/33 virus at an MOI of 5. Cells were harvested at 4.5 hpi and lysed on ice for 10 min in buffer B (10 mM HEPES [PAA catalog no. S11-001], 150 mM NaCl, 0.1% Igepal, 1× Halt protease inhibitor cocktail [Pierce catalog no. 78425]). Debris was removed by centrifugation at 16,200 × *g* for 5 min at 4°C, and lysates were precleared by incubation with streptavidin agarose resin (Pierce catalog no. 20347) for 2 h. Pol II CTD mimic peptides were bound to the beads for 2 h. Peptide-coated beads were washed three times in wash buffer (10 mM HEPES [PAA catalog no. S11-001], 150 mM NaCl, 0.1% Igepal, 1 mM PMSF), blocked with 1% bovine serum albumin for 1 h, and washed twice. Precleared lysates were bound to the peptide-coated beads for 2 h at 4°C, and unbound material was removed by washing three times. The beads were split into two aliquots during the last wash, and they were boiled for 5 min in sample buffer (250 mM Tris-HCl [pH 6.8], 2% SDS, 20 mM DTT, 20% glycerol, 0.01% bromophenol blue) for protein analysis or the RNA was extracted with TRIzol (Ambion), precipitated in the presence of glycogen carrier, and analyzed by primer extension as described above. For detection of viral proteins by Western blot analysis, a custom-made rabbit polyclonal antibody raised against the trimeric influenza virus polymerase (Eurogentec) (24) or a rabbit polyclonal antibody against NP (kind gift from P. Digard) was used.

### Expression and purification of influenza virus polymerase.

For recombinant production of influenza A/WSN/33 virus polymerase in a mammalian cell expression system, HEK 293T cells were grown to about 50% confluence and transfected with pCAGGS-based or pcDNA-based plasmids expressing each of the polymerase subunits (PB1, PA, and C-terminally tandem affinity purfication-tagged PB2) (25, 26) and a plasmid expressing a short 37-nucleotide (nt)-long vRNA-like template derived from segment 5 (27). Cells were harvested at 48 h posttransfection, washed with cold PBS, and lysed on ice for 10 min in buffer C (50 mM HEPES [PAA catalog no S11-001], 200 mM NaCl, 25% glycerol, 0.5% Igepal, 1 mM β-mercaptoethanol, 1 mM PMSF, 1× Halt protease inhibitor cocktail [Pierce catalog no. 78425]). Lysates were subjected to purification on IgG-Sepharose 6 Fast Flow beads (GE Healthcare catalog no. 17-0969-01), and the polymerase was cleaved with AcTEV (Invitrogen) in cleavage buffer (10 mM HEPES [PAA catalog no S11-001], 150 mM NaCl, 10% glycerol, 0.1% Igepal, 1 mM DTT, 1× Halt protease inhibitor cocktail [Pierce catalog no. 78425]). AcTEV contains a His tag that allows its removal by incubation with Ni-nitrilotriacetic acid agarose (Qiagen), and IgG was removed with protein A-Sepharose (Sigma). Recombinant RNA-free influenza A/NT/60/68 and C/Johannesburg/1/66 virus polymerase was produced from baculovirus-infected Sf9 insect cells as described elsewhere (28). To produce the polymerase-vRNA complex, the above protocol was used but with the addition of an RNA-binding step before gel filtration. This was carried out by mixing the polymerase in a high-salt buffer [2 M NaCl, 25 mM HEPES (pH 7.5), 10% glycerol, 1 mM MgCl_2_, 0.1 mM MnCl_2_, 0.5 mM tris(2-carboxyl)phosphine] with a 2- to 3-fold molar excess of two synthetic RNA oligonucleotides corresponding to the vRNA 5′ and 3′ termini (5′-AGUAGAAACAAGGCC-3′ and 5′-GGCCUGCUUUUGCU-3′). The NaCl concentration of the mixture was then reduced to 0.5 M overnight by dialysis. After dialysis, the polymerase-RNA complex was purified away from unbound RNA by gel filtration as described previously (28).

### *In vitro* binding of purified viral polymerase to synthetic Pol II CTD mimic peptides.

Pol II CTD mimic peptides were bound to streptavidin agarose resin as described above for the pulldown assays. Viral polymerase purified from either HEK 293T or Sf9 cells was bound to the peptide-coated beads by incubation for 2 h at 4°C. Complexes were washed three times with wash buffer and split into two aliquots during the last wash. For protein analysis, beads were boiled for 5 min in sample buffer and analyzed by silver staining. For RNA analysis, the RNA in the bound complexes was extracted with TRIzol (Ambion) and precipitated in the presence of glycogen. RNA was dephosphorylated for 10 min at 37°C with FastAP (Fermentas), and the enzyme was inactivated by heating at 75°C for 5 min. Dephosphorylated RNA was 5′ end labeled with [γ-^32^P]ATP for 1 h at 30°C with T4 polynucleotide kinase (Fermentas). Both reactions were carried out in Tango buffer (Fermentas). Labeled RNA was mixed with formamide, heated at 95°C for 3 min, analyzed on 20% polyacrylamide gels containing 7 M urea in Tris-borate-EDTA (TBE) buffer, and visualized by autoradiography.

### *In vitro* transcription assay.

The viral polymerase was immobilized on streptavidin resin coated with Pol II CTD mimic peptides as described above, and its transcriptional activity was evaluated with an [α-^32^P]GTP incorporation assay as previously described (29). Briefly, 1.75 μl of peptide-bound polymerase was incubated in a 3.5-μl reaction volume containing 1 mM ATP, 0.5 mM CTP, 0.5 mM UTP, 1 μCi of [α-^32^P]GTP (PerkinElmer), 10 ng of β-globin mRNA (Sigma), 5 mM MgCl_2_, 2 mM DTT, and 1 U/μl RNasin (Promega) for 2 h at 30°C; mixed with 10 μl of formamide; and heated at 95°C for 3 min. Transcription products were analyzed on 16% polyacrylamide gels containing 7 M urea in TBE buffer and visualized by autoradiography.

## RESULTS

### Viral RNAs coimmunoprecipitate with Pol II.

**FIG 1:**
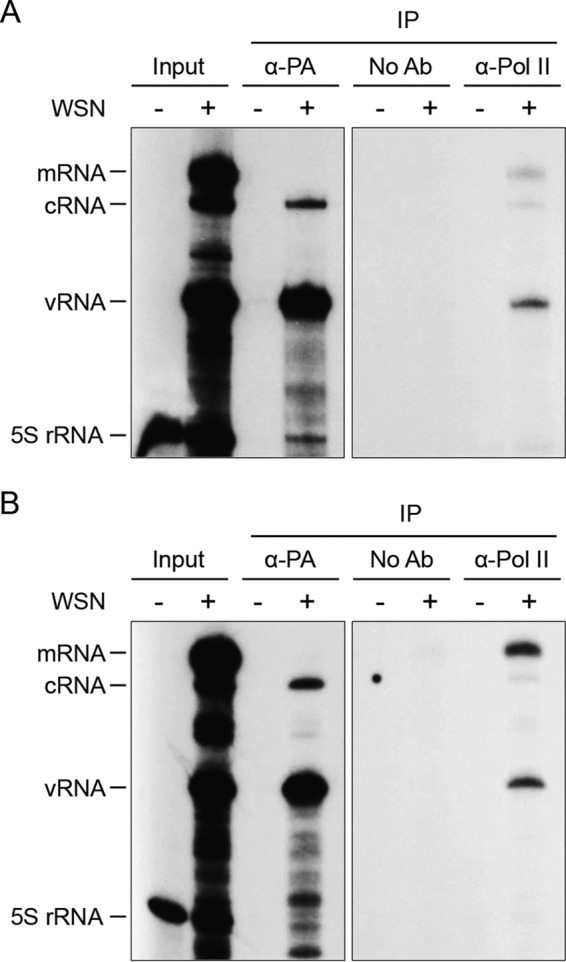
Viral RNAs coimmunoprecipitate with Pol II. HEK 293T cells were infected with influenza A/WSN/33 virus (WSN), harvested at 4.5 hpi, and subjected to RIP. RNAs from cell lysates (input) and immunoprecipitates (IP) were analyzed by primer extension with NA (panel A) and NP (panel B) segment-specific primers. A primer specific for 5S rRNA was used as a control. Note that the sample used for analysis of the input corresponds to 1/10 of that used for the immunoprecipitations. Ab, antibody.

Although the viral polymerase in the absence of NP and vRNA has been shown to interact with Pol II (11), it is unclear whether polymerase in the context of vRNP can also associate with Pol II. If the viral polymerase interaction with Pol II were to facilitate viral mRNA synthesis, viral polymerase in the context of vRNP would bind Pol II. To test this, HEK 293T cells were mock infected or infected with influenza A/WSN/33 virus, harvested at 4.5 hpi, and subjected to RIP. Coimmunoprecipitated RNAs were analyzed for NA (Fig. 1A) and NP (Fig. 1B) viral RNAs by primer extension. As expected, vRNA and cRNA could be immunoprecipitated with an antibody against PA (Fig. 1A and B). When Pol II complexes were analyzed, vRNA and mRNA, as well as small amounts of cRNA, were detected. No 5S rRNA was immunoprecipitated with the PA- or Pol II-specific antibodies, and no RNAs could be detected in a control without primary antibody, confirming the specificity of the interactions. These results suggest that polymerase in the context of vRNPs also interacts with Pol II.

### vRNPs can be pulled down from infected cell lysates with a Pol II CTD mimic peptide phosphorylated on serine-5.

**FIG 2:**
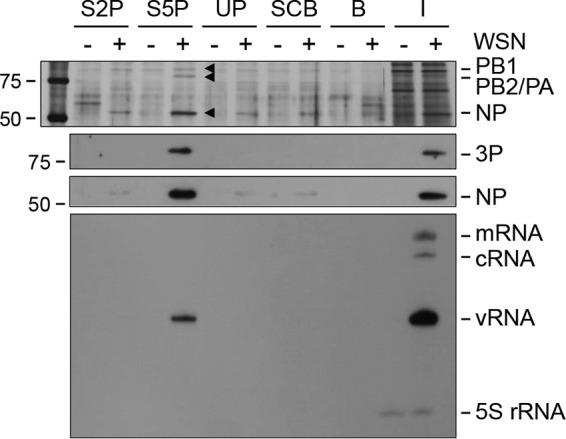
vRNPs from infected cell lysates bind to serine-5-phosphorylated Pol II CTD mimic peptides *in vitro*. HEK 293T cells were infected with influenza A/WSN/33 virus (WSN) or mock infected, harvested at 4.5 hpi, and lysed. Differentially phosphorylated Pol II CTD mimic peptides were immobilized on streptavidin agarose resin and incubated with the lysates. Bound complexes were analyzed by silver staining (top panel), Western blot analysis (middle panels) with antibodies against the viral polymerase (3P) and NP, and primer extension of viral RNAs derived from the NA segment (bottom panel). Background binding to streptavidin agarose resin without peptide was also analyzed, and total cell lysates (input) were included. S2P, Ser2P; S5P, Ser5P; UP, unphosphorylated; SCB, scrambled; B, beads; I, input. The values to the left are molecular sizes in kilodaltons.

To further investigate the interaction between vRNPs and Pol II, a peptide pulldown assay was developed. Biotinylated Pol II CTD mimic peptides containing four copies of the conserved heptapeptide repeat (YSPTSPS) of the Pol II CTD were chemically synthesized. Although the full-length human Pol II CTD consists of 52 heptad repeats, we reasoned that 4 repeats would be sufficient for the interaction, on the basis of structural studies of other CTD-binding proteins (30). It was previously shown that the interaction of the viral polymerase with Pol II depends on the phosphorylation status of the CTD. In particular, the viral polymerase interacts with the initiating form of Pol II, which is phosphorylated on serine-5 in the CTD, but not with the elongating form, phosphorylated on serine-2 (11). Therefore, we used a peptide phosphorylated on serine-2 (Ser2P) or serine-5 (Ser5P), an unphosphorylated peptide, and a scrambled control peptide (Table 1). The results show that the peptide phosphorylated on serine-5 was able to pull down vRNPs from infected cell lysates, as indicated by the presence of viral polymerase and NP in the bound complexes (Fig. 2). The peptide phosphorylated on serine-2, the unphosphorylated peptide, and the scrambled control peptide bound only background levels of NP, while no viral polymerase could be detected. Furthermore, RNA extracted from the complexes bound to the peptides was analyzed by primer extension with a primer specific for the NA segment RNAs. The peptide phosphorylated on serine-5 was able to specifically pull down NA vRNA (Fig. 2). No mRNA or cRNA was detected. Taken together, these data further support the notion that viral polymerase in the context of vRNPs can interact with Pol II.

### Influenza A virus polymerase binds directly and specifically to Pol II CTD phosphorylated on serine-5.

**FIG 3:**
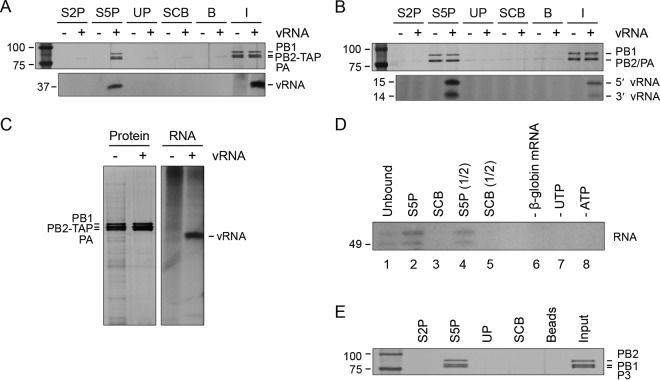
Purified recombinant influenza virus polymerase binds to serine-5-phosphorylated Pol II CTD mimic peptides *in vitro*. (A) Recombinant viral polymerase from influenza A/WSN/33 (H1N1) virus was expressed in and purified from HEK 293T cells in the presence (+) or absence (−) of a 37-nt-long vRNA-like template. Peptides mimicking different phosphorylation states of the Pol II CTD (Ser2P, Ser5P, unphosphorylated) and a scrambled control peptide were immobilized on streptavidin agarose resin and incubated with purified viral polymerase. Complexes bound to the peptides were analyzed by silver staining (top), and RNA was detected by 5′-end labeling with [γ-^32^P]ATP (bottom). (B) Recombinant viral polymerase from influenza A/NT/60/68 (H3N2) virus was expressed in and purified from Sf9 insect cells in the presence (+) or absence (−) of 15- and 14-nt-long RNAs corresponding to the 5′ and 3′ ends of the vRNA promoter, respectively. The polymerase was incubated with the Pol II CTD mimic peptides, and bound complexes were analyzed as described for panel A. (C) Input samples of recombinant viral polymerase from panel A analyzed for the presence of contaminating host proteins and RNA. Silver staining shows higher levels of contaminating host proteins copurifying with the viral polymerase if vRNA is absent. Labeling of RNA with [γ-^32^P]ATP shows that, in the absence of vRNA, higher levels of contaminating cellular RNA are present, as represented by the strong smear. (D) *In* *vitro* transcription by recombinant viral polymerase from influenza A/WSN/33 (H1N1) virus expressed in and purified from HEK 293T cells in the presence of a 37-nt-long vRNA-like template, with β-globin mRNA as a cap donor. Transcription products of input polymerase are shown as a positive control (lane 1). Lanes 2 to 5 show the transcriptional activity of the polymerase captured by Pol II CTD mimic peptides immobilized on streptavidin agarose resin. Transcription products are synthesized when the polymerase is bound to a Pol II CTD mimic peptide phosphorylated on serine-5 (lanes 2 and 4). A scrambled peptide with no detectable polymerase bound is included as a negative control (lanes 3 and 5). No transcription products are obtained in the absence of the β-globin mRNA cap donor, UTP, or ATP (lanes 6 to 8). Lanes 4 and 5 show the result obtained with a 2-fold dilution of the viral polymerase, compared to that in lanes 2 and 3. (E) Influenza C/Johannesburg/1/66 virus recombinant polymerase was expressed in and purified from Sf9 insect cells. The polymerase was incubated with the peptides as described above, and bound complexes were analyzed by silver staining. S2P, Ser2P; S5P, Ser5P; UP, unphosphorylated; SCB, scrambled; B, beads; I, input. The values to the left of the panels are molecular sizes in kilodaltons for protein panels and numbers of nucleotides for RNA panels.

To address the question of whether the viral polymerase interacts directly with the Pol II CTD, the set of peptides described above was incubated with recombinant viral polymerase expressed in and purified from HEK 293T cells in the presence or absence of a 37-nt-long vRNA-like template. Short vRNA-like templates can be transcribed and replicated in cells in the absence of NP (23, 31). The viral polymerase bound specifically to the serine-5-phosphorylated CTD mimic peptide and did so only when vRNA was coexpressed (Fig. 3A, top). To confirm that the vRNA template copurified with the polymerase remained bound throughout the peptide binding assay, RNA was extracted from bound complexes, 5′ end labeled with [γ-^32^P]ATP, and analyzed on a polyacrylamide gel. As expected, the 37-nt-long vRNA was present in the complexes bound to the CTD mimic peptide phosphorylated on serine-5 (Fig. 3A, bottom). Only background binding of the polymerase to the other peptides was observed.

As we could not entirely exclude the possibility that a factor copurifying from mammalian cells with the viral polymerase was involved in mediating the interaction with the CTD mimic peptide, we also tested viral polymerase produced in Sf9 insect cells in the presence or absence of 15- and 14-nt-long RNA oligonucleotides mimicking the 5′ and 3′ ends of the vRNA promoter. As expected, these preparations of the viral polymerase also bound specifically to the CTD mimic peptide phosphorylated on serine-5. However, in the case of these highly pure preparations, the presence or absence of vRNA did not affect binding, as both the RNA-free and RNA-bound forms interacted equally well (Fig. 3B, top). We confirmed that the 5′ and 3′ ends of the vRNA promoter remained bound to the polymerase that bound to the Pol II CTD mimic peptide phosphorylated on serine-5 (Fig. 3B, bottom). The differential requirement for a vRNA promoter in CTD peptide binding by insect and mammalian cell-derived viral polymerase may be due to the presence of contaminating host factors (either protein or RNA). In the mammalian system, we consistently find that coexpression of short vRNA-like templates reduces the amount of cellular proteins and RNA that copurifies with the viral polymerase (Fig. 3C). Therefore, the inability of viral polymerase expressed in mammalian cells without vRNA to bind to a CTD peptide may be due to higher levels of contaminating inhibitory cellular factors. Altogether, these data show that the binding of the viral polymerase to the serine-5-phosphorylated CTD of Pol II is direct and both the RNA-free form and the vRNA promoter-bound form of the viral polymerase are able to bind Pol II.

### Influenza A virus polymerase is transcriptionally active when bound to a Pol II CTD mimic peptide.

If the binding of the viral polymerase to the CTD of Pol II were to facilitate cap snatching for viral transcription, polymerase bound to the Pol II CTD would be expected to be active in transcription. To test this hypothesis, recombinant viral polymerase was purified from HEK 293T cells coexpressing a 37-nt-long vRNA-like template and immobilized on streptavidin resin coated with a CTD mimic peptide phosphorylated on serine-5. The activity of the viral polymerase was tested *in vitro* with β-globin mRNA as a cap donor to prime viral transcription. Quantification of capped transcription products revealed that at least 35-fold higher activity was obtained with the polymerase bound to the CTD mimic peptide phosphorylated on serine-5 than with the negative-control scrambled peptide (Fig. 3D). Only background levels of products were obtained if the cap donor, UTP, or ATP was omitted from the reaction mixture. These results show that binding of the viral polymerase to the Pol II CTD is compatible with viral transcription.

### Binding of the viral polymerase to the Pol II CTD is conserved in evolutionarily distant influenza C virus.

If the interaction of the viral polymerase with Pol II CTD were required for viral transcription, it would be expected that this interaction would also occur with evolutionarily distant influenza viruses such as influenza C virus. To test this hypothesis, recombinant influenza C virus RNA polymerase was expressed in and purified from Sf9 cells in the absence of vRNA and incubated with the set of CTD mimic peptides as described above. Influenza C virus polymerase bound specifically to the CTD mimic peptide phosphorylated on serine-5 (Fig. 3E), matching the binding pattern found for influenza A virus polymerase (Fig. 3A and B). This result shows that the interaction of the influenza virus polymerase with Pol II is evolutionarily conserved across influenza virus genera.

## DISCUSSION

In this study, we investigated the molecular details of how the influenza virus transcriptional machinery interacts with cellular Pol II. We provide biochemical evidence that not only RNA-free trimeric viral polymerase but also viral polymerase in the context of vRNPs can associate with Pol II. First, we showed that viral RNAs are present in complexes containing Pol II. Second, we were able to pull down vRNPs from influenza virus-infected cell lysates with Pol II CTD mimic peptides. We showed that viral polymerase, NP, and vRNA were present in the pulldowns. vRNPs were pulled down specifically only with a Pol II CTD mimic peptide phosphorylated on serine-5, in agreement with previous data showing that the viral polymerase associates with the serine-5-phosphorylated form of the CTD (11).

In addition to demonstrating that vRNPs can interact with the CTD of Pol II, we also show here that the binding between the viral polymerase and the CTD is direct. Indeed, recombinant trimeric influenza A virus polymerase from two different viral strains (A/WSN/33 and A/NT/60/68, purified from mammalian and insect cell expression systems, respectively) was able to bind specifically to a Pol II CTD mimic peptide that was phosphorylated on serine-5. We were not able to detect any binding when polymerase subunits were individually expressed and purified (data not shown). This result is in agreement with previous data showing that none of the individually expressed polymerase subunits, or combinations of two subunits, copurified with a tagged version of the Pol II CTD (11). However, this may be because individually expressed and purified viral polymerase subunits are misfolded or are in a conformation incompatible with CTD binding. Indeed, a yeast two-hybrid screen identified the PA subunit as an interactor with the large subunit of Pol II (14).

In terms of evolution, influenza A and B viruses are more closely related to each other than they are to influenza C virus (32, 33). In fact, amino acid sequences of the polymerase subunits show the least conservation between influenza A and C viruses (38.4, 23.3, and 25.4% identity for PB1, PB2, and PA/P3, respectively) (34). Therefore, we chose the viral polymerase of influenza C virus to test whether polymerase binding to the CTD of Pol II is a conserved feature of influenza viruses. Indeed, our results show that influenza C virus polymerase binds directly to the initiating form of Pol II, which suggests that influenza viruses have evolved a conserved mechanism to hijack the transcriptional machinery of the host cell. Hence, the interaction domain of the influenza virus RNA polymerase involved in binding to the CTD of Pol II is likely to be highly conserved between influenza virus genera, and therefore, drugs targeting this interaction domain could be active against different influenza virus types.

**FIG 4:**
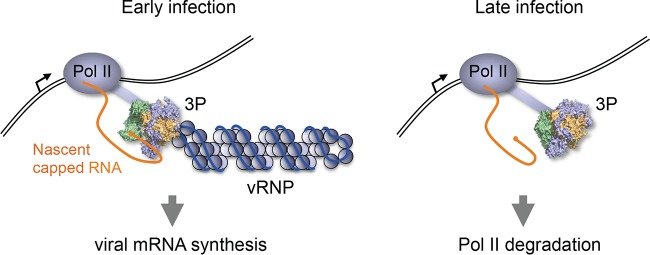
Model of the dual roles of the interaction of the influenza virus RNA polymerase with the CTD of the large subunit of Pol II. Early in infection (left), binding of the viral polymerase in the context of vRNP to the Pol II CTD facilitates cap snatching from nascent host capped RNA. The viral polymerase (3P) is shown in a surface model representation in the “transcription preinitiation” state with the PB2 cap-binding and PA endonuclease domains aligned for cap snatching (Protein Data Bank [PDB] code 4WSB). Late in infection (right), binding of the free viral polymerase (3P) to the Pol II CTD triggers Pol II degradation. The viral polymerase is shown in the apo conformation, with the cap-binding pocket of PB2 blocked (PDB code 5D98). PB1, dark yellow; PB2, green; PA/P3, blue.

Our data show that both RNA-free viral polymerase and vRNPs associate with the CTD of Pol II. The association of vRNPs with the CTD likely provides the viral polymerase with a platform to carry out transcription, enabling the polymerase to access nascent host capped RNAs, as well as splicing factors and factors required for mRNP assembly (35). The CTD of Pol II is dynamically modified during the transcription cycle, undergoing different phosphorylation states that correlate with Pol II progress through transcription. Thus, a hypophosphorylated CTD is a mark of preinitiating Pol II that can bind at promoters, while serine-5 and serine-2 phosphorylation marks correspond to initiating and elongating Pol II, respectively (36, 37). Influenza virus vRNPs specifically target the Pol II CTD when it is phosphorylated at serine-5, the form of Pol II that is involved in the capping of nascent transcripts. Therefore, the physical association of influenza vRNPs with Pol II early in infection is likely to promote cap snatching by providing access to nascent cellular capped RNAs for viral mRNA synthesis (Fig. 4). The regulation of the interaction between the viral polymerase in the context of vRNPs and the CTD remains unclear. Thus, it is not known whether the viral polymerase is released from the CTD immediately after cap snatching or remains associated with it while it completes mRNA synthesis.

What would be the function of RNA-free trimeric polymerase associating with Pol II? Such a polymerase, lacking template vRNA, would not be competent in cap snatching, as the polymerase needs to be associated with both the 5′ and 3′ ends of the vRNA to cap snatch efficiently (38–40). Influenza virus infection results in degradation of the large subunit of Pol II at late stages of infection, and expression of the polymerase in the absence of vRNA and NP has been shown to induce Pol II degradation (17). Expression of all three subunits of the viral polymerase was required for Pol II degradation. Neither the expression of individual polymerase subunits nor the expression of combinations of two of them was sufficient to induce Pol II degradation, in agreement with the finding that all three subunits of the viral polymerase are required for Pol II interaction. Indeed, the ability of the polymerase to induce degradation of Pol II has been linked to its ability to interact with Pol II (18). Thus, we speculate that, late in infection, as free viral polymerase accumulates in the infected host cell nucleus, the main role of binding of the polymerase to Pol II is to trigger Pol II degradation to inhibit host gene expression, including the expression of antiviral genes (Fig. 4). In fact, the ability of the viral polymerase to degrade Pol II has been linked to virulence (41). This model is consistent with the pattern of the accumulation of the different types of viral RNAs in infected cells. mRNA synthesis peaks early in infection; this is followed by a sharp decline late in infection, most likely because of the exhaustion of a source of capped RNA primers. In contrast, vRNA replication, which is independent of Pol II, continues late into infection.

It is not clear how binding of the polymerase to the CTD of Pol II would trigger Pol II degradation. However, the ubiquitin-proteasome system is likely to be involved. Our group reported that increasing amounts of ubiquitylated Pol II are present late in infection and the expression of the viral polymerase trimer is sufficient to trigger ubiquitylation of the serine-5-phosphorylated form of Pol II. Furthermore, the expression of a mutant viral polymerase with reduced Pol II-binding activity induced reduced levels of ubiquitylated Pol II (18). We also found that the viral polymerase interacts with several ubiquitin ligases (42). It is possible that the influenza virus polymerase, by binding the CTD of Pol II late in infection, recruits a ubiquitin ligase to mediate the ubiquitylation of Pol II and its subsequent degradation by the proteasome. Although this mechanism would lead to the specific degradation of serine-5-phosphorylated Pol II, given the dynamic nature of CTD phosphorylation, other forms of Pol II would be depleted as well. Indeed, specific reduction of the hypophosphorylated form of Pol II has been reported in virus-infected cells and also upon the expression of the viral polymerase heterotrimer (17, 18). The influenza virus polymerase has been shown to exist in multiple conformations, depending on vRNA binding (34, 40, 43, 44). The vRNP-bound polymerase associated with Pol II involved in cap snatching would be in the conformation described for the influenza A and B virus polymerases. However, the RNA-free polymerase triggering Pol II degradation might be in the apo conformation described for the influenza C virus polymerase. Only the apo conformation might be competent in recruiting ubiquitin ligases such that no degradation would occur as a result of viral polymerase binding in the context of vRNPs.

Induction of Pol II degradation is not unique to influenza virus. La Crosse and Schmallenberg viruses, both members of the family Bunyaviridae, encode the NSs protein that is known to trigger a DNA damage response-like degradation of transcribing RNA Pol II (45, 46). Perhaps the influenza virus RNA polymerase also acts by triggering a DNA damage response-like phenomenon.

Taken together, the results of this study show that both vRNP-bound and free RNA polymerases associate with Pol II and we propose that the two associations have different roles during the viral replication cycle (Fig. 4). On the one hand, this interaction allows the virus to promote the transcription of its genes; on the other, it allows the virus to shut off the host, with important consequences for virulence.
